# Triglyceride–glucose index and the incidence of atherosclerotic cardiovascular diseases: a meta-analysis of cohort studies

**DOI:** 10.1186/s12933-021-01268-9

**Published:** 2021-04-03

**Authors:** Xiaobo Ding, Xiaozhen Wang, Jing Wu, Manli Zhang, Meizi Cui

**Affiliations:** 1 Radiology Department, The First Hospital of Jilin University, Changchun, 130021 China; 2 Department of Breast Surgery, The First Hospital of Jilin University, Changchun, 130021 China; 3 Department of General Practice, The First Hospital of Jilin University, Changchun, 130021 China; 4 Department of Hepatology and Gastroenterology, The Second Part of First Hospital, Jilin University, Changchun, 130021 China; 5 Department of Cadre Ward, The First Hospital of Jilin University, No.1 Xinmin Street, Changchun, 130021 China

**Keywords:** Triglyceride-glucose index, Insulin resistance, Atherosclerotic cardiovascular diseases, Coronary artery disease, Meta-analysis

## Abstract

**Background:**

Insulin resistance has been demonstrated to be involved in the pathogenesis of atherosclerotic cardiovascular diseases (ASCVDs). This study evaluated the association between the triglyceride–glucose (TyG) index, a novel surrogate indicator of insulin resistance, and the incidence of ASCVDs in people without ASCVDs at baseline by performing a meta-analysis.

**Methods:**

Cohort studies reporting the multivariate-adjusted association between the TyG index and the incidence of ASCVDs were obtained by searching the PubMed and Embase databases. A random-effects model incorporating intra-study heterogeneity was applied to combine the results.

**Results:**

Eight cohort studies comprising 5,731,294 participants were included in this meta-analysis. The results showed that compared to those with the lowest TyG index category, participants with the highest TyG index category were independently associated with a higher risk of ASCVDs [hazard ratio (HR): 1.61, 95% confidence interval (CI) 1.29–2.01, I^2^ = 80%, P < 0.001]. This finding was consistent with the meta-analysis results with the TyG index analyzed as a continuous variable (HR per 1-unit increment of the TyG index: 1.39, 95% CI 1.18–1.64, I^2^ = 89%, P < 0.001). Subgroup analyses suggested that the age, sex, and diabetic status did not significantly affect the association (for subgroup analyses, all P > 0.05). Moreover, participants with the highest TyG index category were independently associated with a higher risk of coronary artery disease [(CAD), HR: 1.95, 95% CI 1.47–2.58, I^2^ = 92%, P < 0.001] and stroke (HR: 1.26, 95% CI 1.23–1.29, I^2^ = 0%, P < 0.001).

**Conclusions:**

A higher TyG index may be independently associated with a higher incidence of ASCVDs, CAD, and stroke in people without ASCVDs at baseline.

## Background

Despite significant advances in the prevention and treatment of cardiovascular diseases, atherosclerotic cardiovascular diseases (ASCVDs), which mainly include coronary artery disease (CAD) and stroke, remain one of the leading causes of death worldwide [1, 2]. Established risk factors of ASCVDs include age, the male sex, family history of ASCVDs, obesity, hypertension, hypercholesteremia, and diabetes [3, 4]. However, more recent studies have demonstrated that some patients without these risk factors may still develop ASCVDs, thus highlighting the importance of identifying novel risk factors for ASCVDs in the general population [5–7]. Previous studies have suggested that insulin resistance, which is not only prevalent in patients with type 2 diabetes mellitus but also in people with obesity or metabolic syndrome, may also be involved in the pathogenesis of ASCVDs [8–10]. Classically, the “gold standard” method for the evaluation of insulin sensitivity is the hyperinsulinemic-euglycemic clamp test [11]. However, this method is time consuming and expensive, which limit its use in clinical settings [12]. Interestingly, the triglyceride–glucose (TyG) index, a parameter derived from the fasting blood glucose and triglyceride levels, has been proposed as a convincing indicator of insulin resistance [13]. Observational studies have shown that a higher TyG index is associated with the prevalence of ASCVDs in the general population [14]. Nevertheless, these studies mostly consist of a cross-sectional design [15–17]. Recently, accumulating cohort studies evaluating the association between the TyG index at baseline and the subsequent incidence of ASCVDs in the general population have been published [18–25]. Accordingly, the aim of this study was to summarize the potential independent association between the TyG index and the risk of ASCVDs in participants without ASCVDs at baseline.

## Methods

The Meta-analysis of Observational Studies in Epidemiology [26] Statement and Cochrane’s Handbook [27] were followed for the design, performance, and reporting of this meta-analysis.

### Literature search

Electronic databases including PubMed and Embase were searched with the combination of the following terms: (1) “TyG index” OR “triglyceride-glucose index” OR “triglyceride and glucose index” OR “triglyceride glucose index” OR “triacylglycerol glucose index;” and (2) “atherosclerotic cardiovascular disease” OR “ASCVD” OR “cardiovascular events” OR “MACE” OR “cardiovascular” OR “coronary artery disease” OR “CAD” OR “CHD” OR “stroke.” Filters were applied so that only studies conducted in humans and published in English or Chinese were considered. The reference lists of related original and review articles were manually searched for potential eligible studies. The final literature search was performed on January 5, 2021.

### Study selection

Studies fulfilling all of the following criteria were included: (1) cohort studies published as full-length articles; (2) included an adult population without ASCVDs at baseline; (3) TyG index was measured at baseline; (4) the outcome of interest was CAD, stroke, or a composite outcome of ASCVD; and (5) reported the relative risk for the association after adjustment of potential confounding factors. The TyG index was calculated as ln[TG (mg/dL) × FPG (mg/dL)/2] [28]. A composite outcome of ASCVDs was defined as the incidence of CAD, stroke, and peripheral artery disease (PAD). The diagnosis of CAD or stroke was consistent with the criteria of the original studies. Typically, CAD was defined as acute myocardial infarction, angina pectoris, and other ischemic heart disease, both fatal and non-fatal. Stroke was defined as fatal and non-fatal ischemic stroke.

### Data extraction and quality evaluation

The literature search, data extraction, and quality assessment of the included studies were performed by two authors independently, according to the predefined criteria. Discrepancies were resolved by consensus. The extracted data were as follows: (1) name of the first author, publication year, and country; (2) study design characteristics; (3) participant characteristics, including health status, sample size, age, sex, and proportion of patients with diabetes; (4) patterns for TyG index analysis; (5) follow-up duration; (6) outcomes reported and methods for outcome validation; and (7) confounding factors adjusted in the multivariate analyses. The quality of each study was evaluated using the Newcastle–Ottawa Scale [29]. This scale ranges from 1 to 9 in total and judges the quality of cohort studies according to the selection of the study groups, comparability of the groups, and ascertainment of the outcome of interest.

### Statistical analyses

Hazard ratios (HRs) and their corresponding 95% confidence intervals (CIs) were applied as the general measure for the association between the TyG index and ASCVDs, CAD, and stroke in an adult population without ASCVDs at baseline. For studies with the TyG index analyzed as a categorical variable, the HRs of the ASCVD incidence in participants with the highest TyG index level compared to those with the lowest TyG index level were extracted. For studies with the TyG index analyzed as a continuous variable, the HRs of the ASCVD incidence per 1-unit increment of the TyG index was extracted. Data of the HRs and their standard errors were calculated from the 95% CIs or P values; then they were logarithmically transformed for variance stabilization and distribution normalization [27]. Cochran’s Q test was used to evaluate the heterogeneity among the included cohort studies as well as to estimate the I^2^ statistic [30]. If I^2^ > 50%, a significant heterogeneity was considered. A random-effects model was used to synthesize the HR data because this model is considered as a more generalized method to incorporate the potential heterogeneity among included studies [27]. Sensitivity analyses, which excluded one individual study at a time, were performed to test the stability of the results [31]. Predefined subgroup analyses were carried out to evaluate the influences of the study characteristics, including age, sex, and diabetic status, on the association between the TyG index and the ASCVD risk. The potential publication bias was assessed by visual inspection of the funnel plots for symmetry as well as by Egger’s regression asymmetry test [32]. RevMan (Version 5.1; Cochrane Collaboration, Oxford, UK) and STATA (Version 12.0) software were used to perform the meta-analysis and statistical analysis.

## Results

### Literature search

**Fig. 1:**
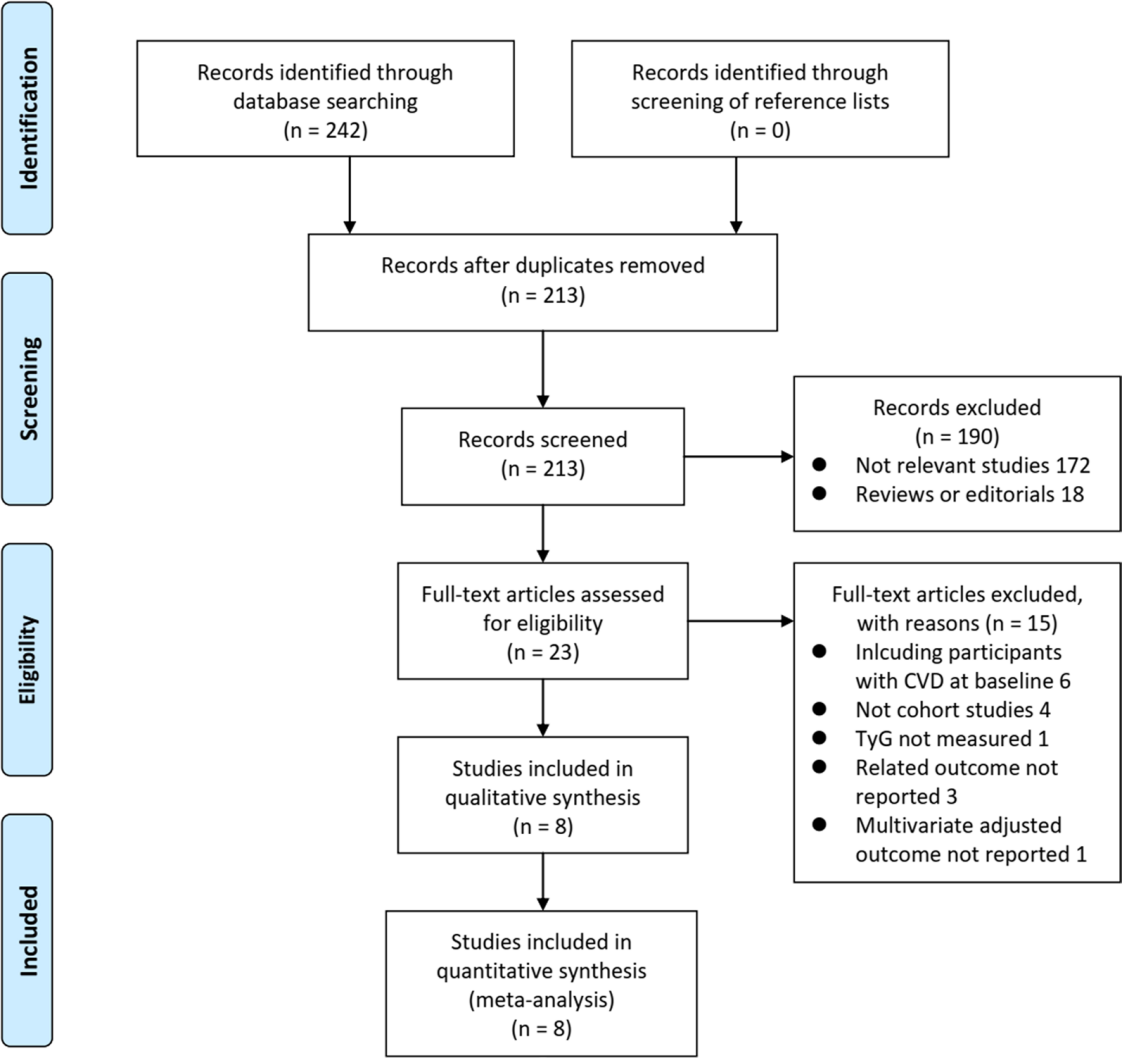
Flowchart of the database search and study identification

Figure 1 shows the process of the database search. Briefly, 213 articles were obtained via the initial literature search of the PubMed and Embase databases after excluding the duplications. Among them, 190 articles were excluded through screening of the titles and abstracts for irrelevance. Subsequently, 23 articles underwent full-text review. Of these, 15 articles were further excluded for the reasons listed in Fig. 1. Finally, eight cohort studies were obtained for this meta-analysis [18–25].

### Study characteristics and quality evaluation

The characteristics of the included studies are summarized in Table 1. Overall, eight cohort studies [18–25] comprising a total of 5,731,298 participants who did not have any ASCVDs at baseline were included in this meta-analysis. The studies were performed in Spain [19], Argentina [20], China [21, 25], Korea [23, 24], and Iran [22]. Populations such as first-time attendee outpatients to an internal medicine department [19], participants in a routine health check-up program [18], community populations [20, 22–25], and diabetes mellitus patients without any ASCVDs [21] were included. The sample sizes of the included studies varied between 723 and 5,593,134. The mean ages of the included participants varied from 46 to 71 years old, with the proportion of male participants ranging from 32 to 80%. The baseline TyG index was analyzed as a categorical variable in four studies [19, 22–25], as a continuous variable in one study [21], and as both in three studies [18, 20, 22]. The follow-up durations varied between 2.4 and 16.1 years. The incidence of ASCVD outcomes was validated by medical record review in one study [21], by clinical evaluation in two studies [20, 22], and by International Classification of Diseases 10 codes in the other five studies [18, 19, 21, 24, 25]. Age, sex, body mass index, smoking status, blood pressure, serum total cholesterol or low-density lipoprotein cholesterol, diabetic status, and concurrent antihypertensive or lipid-lowering medications were adjusted to a varying degree when the association between the TyG index and the ASCVD risk was reported. The Newcastle–Ottawa Scale score was nine for all of the included studies, indicating good study quality (Table 2).

**Table 1:** Characteristics of the included cohort studies

Study	Country	Design	Characteristics of participants	Number of participants	Mean age (years)	Male (%)	DM (%)	TyG index analysis	Follow-up duration (years)	Outcome validation	Outcomes reported	Variables adjusted
Sanchez-Inigo [19]	Spain	PC	First-time attendee outpatients to an internal medicine department without ASCVDs	5,014	54.4	61.2	5.2	Q5:Q1	8.8	ICD-10	Composite ASCVDs (505), CAD (233), stroke (157), and PAD (74)	Age, sex, BMI, smoking, alcohol intake, lifestyle pattern, HTN, T2DM, antiplatelet therapy, HDL-C, and LDL-C
Salazar [20]	Argentina	PC	Community population without DM or ASCVDs	723	50.3	32.8	0	Q4:Q1–Q3; Continuous	8.2	Clinical evaluation	Composite ASCVDs (42)	Age, sex, smoking, LDL-C, BMI, and aspirin, antihypertensive and lipid-lowering drug use
Su [21]	China	RC	T2DM patients without previous ASCVDs	3,524	61.7	49.1	100	Continuous	5.9	Medical record review	Composite ASCVDs (215)	Age, sex, HTN, BMI, HDL-C, eGFR, antihypertensive and lipid-lowering drug use
Li [18]	China	RC	Participants aged over 60 years without previous ASCVDs who participated in a routine health check-up program	6,078	70.5	53.1	11.8	Q4:Q1; Continuous	5.5	ICD-10	Composite ASCVDs (705), CAD (500), and stroke (234)	Age, sex, living alone, current smoker, alcohol consumption, exercise, BMI, SBP, HDL-C, LDL-C, and T2DM
Hong [23]	Korea	RC	Community population without ASCVDs	5,593,134	53.0	50.5	3.7	Q4:Q1	8.2	ICD-10	Composite ASCVDs (146,744), CAD (62,577), and stroke (89,120)	Age, sex, smoking, alcohol consumption, regular physical activity, low socioeconomic status, BMI, HTN, and TC
Park [24]	Korea	PC	Community population without DM or ASCVDs	16,455	46.1	51.2	0	Q4:Q1	2.4	ICD-10	CAD (322)	Age, sex, BMI, smoking status, alcohol intake, physical activity, mean arterial BP, hs-CRP, CKD, and hypertension medication
Barzegar [22]	Iran	PC	Community population without ASCVDs	7,521	46.7	44.7	13.2	Q4:Q1; Continuous	16.1	Clinical evaluation	Composite ASCVDs (1084), and CAD (924)	Age, sex, WC, BMI, educational level, smoking status, physical activity, family history of CVD, T2DM, HTN, LDL-C, HDL-C, and lipid-lowering drugs
Tian [25]	China	PC	Community population without ASCVDs	98,849	51.8	79.8	3.07	Q4:Q1	11.0	ICD-10	CAD (1555)	Age, sex, education, income, smoking, alcohol abuse, physical activity, BMI, SBP, DBP, HTN, DM, dyslipidemia, antidiabetic drugs, lipid-lowering drugs, antihypertensive drugs, and HDL-C, LDL-C, and hs-CRP levels at baseline

TyG: triglyceride–glucose; DM: diabetes mellitus; PC: prospective cohort; RC: retrospective cohort; T2DM: type 2 diabetes mellitus; ASCVDs: atherosclerotic cardiovascular disease; CVD: cardiovascular disease; ICD-10: International Classification of Diseases, tenth edition; CAD: coronary artery disease; PAD: peripheral artery disease; HTN: hypertension; HDL-C: high-density lipoprotein cholesterol; LDL-C: low-density lipoprotein cholesterol; BMI: body mass index; eGFR: estimated glomerular filtrating rate; SBP: systolic blood pressure; BP: blood pressure; DBP: diastolic blood pressure; TC: total cholesterol; hs-CRP: high-sensitivity C-reactive protein; CKD: chronic kidney disease

**Table 2:** Details of quality evaluation via the Newcastle–Ottawa Scale

Study	Representativeness of the exposed cohort	Selection of the non-exposed cohort	Ascertainment of exposure	Outcome not present at baseline	Control for age	Control for other confounding factors	Assessment of outcome	Sufficient follow-up duration	Adequacy of follow-up of cohorts	Total
Sanchez-Inigo [19]	1	1	1	1	1	1	1	1	1	9
Salazar [20]	1	1	1	1	1	1	1	1	1	9
Su [21]	1	1	1	1	1	1	1	1	1	9
Li [18]	1	1	1	1	1	1	1	1	1	9
Hong [23]	1	1	1	1	1	1	1	1	1	9
Park [24]	1	1	1	1	1	1	1	1	1	9
Barzegar [22]	1	1	1	1	1	1	1	1	1	9
Tian [25]	1	1	1	1	1	1	1	1	1	9

### TyG index and the incidence of ASCVDs

**Fig. 2:**
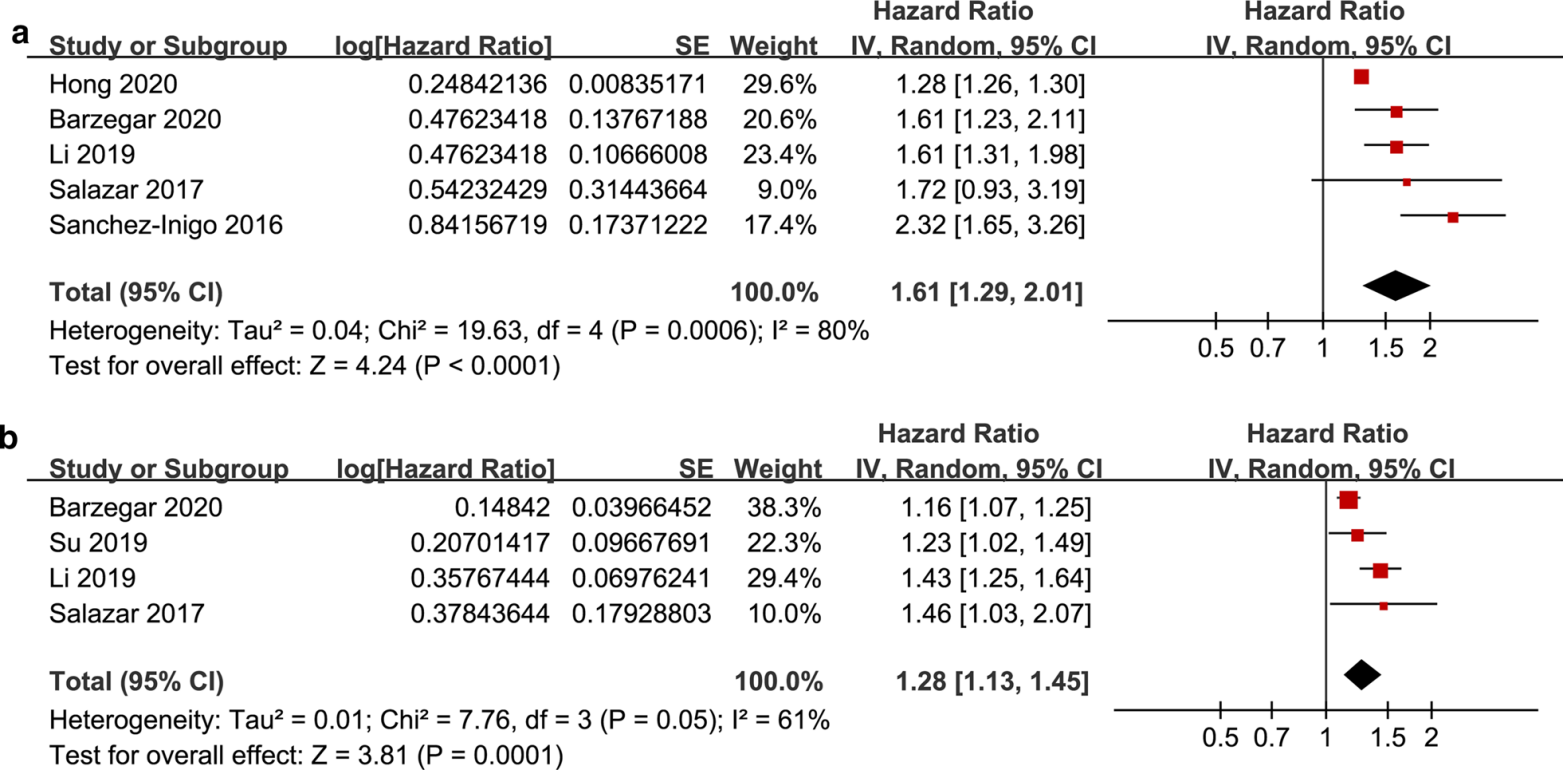
Forest plots for the meta-analysis of the association between the TyG index and the risk of ASCVDs. **a** Meta-analysis with the TyG index analyzed as a categorical variable. **b** Meta-analysis with the TyG index analyzed as a continuous variable

**Fig. 3:**
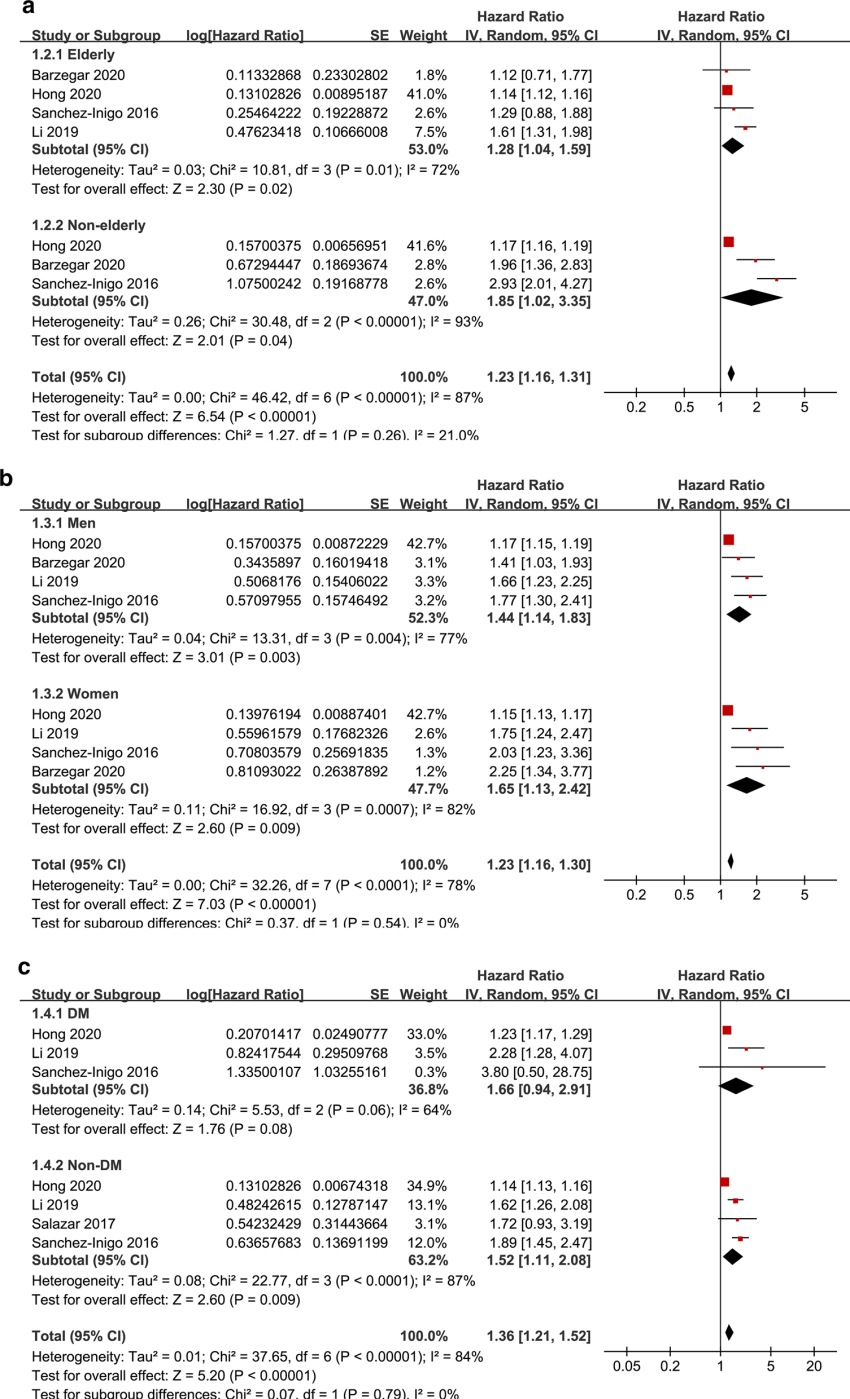
Subgroup analyses for the association between the TyG index analyzed as a categorical variable and the risk of ASCVDs. **a** Subgroup analysis according to the age of the participants. **b** Subgroup analysis according to the sex of the participants. **c** Subgroup analysis according to the diabetic status of the participants

The pooled results of five studies [18–20, 22, 23] showed that compared to participants with the lowest TyG index category at baseline, the participants with the highest TyG index category had a significantly increased risk of ASCVD during follow-up (HR: 1.61, 95% CI 1.29–2.01, I^2^ = 80%, P < 0.001; Fig. 2a). This finding was consistent with the meta-analysis results with the TyG index analyzed as a continuous variable (four studies [18–22], HR per 1-unit increment of the TyG index: 1.28, 95% CI 1.13–1.45, I^2^ = 61%, P < 0.001; Fig. 2b). The sensitivity analyses by excluding one study at a time showed similar results (HRs for the TyG index analyzed as a categorical variable: 1.46–1.74, all P < 0.05; HRs for the TyG index analyzed as a continuous variable: 1.18–1.37, all P < 0.05). The subgroup analyses showed that the participants with the highest TyG index category had a significantly increased risk of ASCVDs compared to those with the lowest category and were independent of the age, sex, or diabetic status of the participants (for subgroup analyses, all P > 0.05; Fig. 3a–c).

### TyG index and the incidence of CAD and stroke

**Fig. 4:**
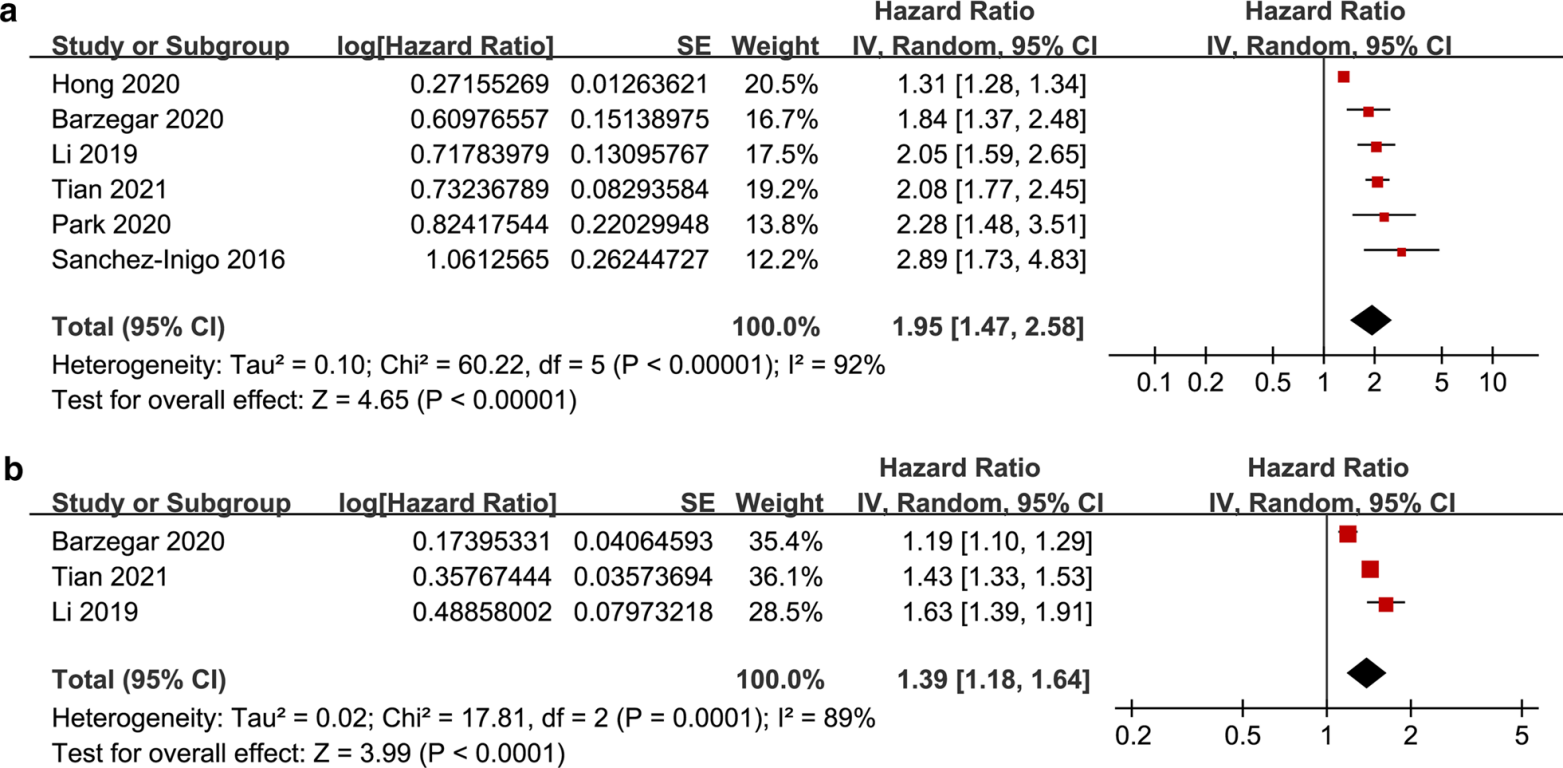
Forest plots for the meta-analysis of the association between the TyG index and the risk of CAD. **a** Meta-analysis with the TyG index analyzed as a categorical variable. **b** Meta-analysis with the TyG index analyzed as a continuous variable

**Fig. 5:**
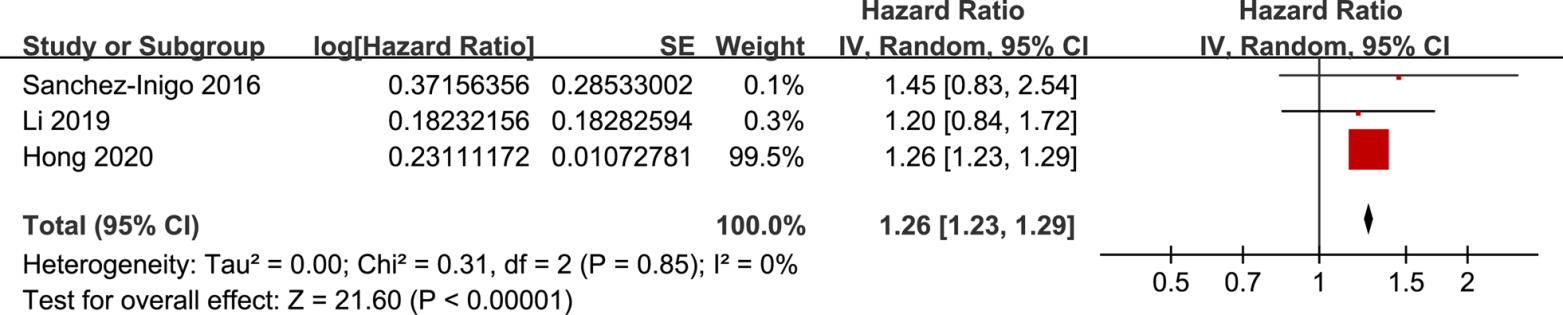
Forest plots for the meta-analysis of the association between the TyG index analyzed as a categorical variable and the risk of stroke

The pooled results of six studies [18, 19, 22–25] showed that the participants with the highest TyG index category had a significantly increased risk of CAD during follow-up compared to those with the lowest TyG category (HR: 1.95, 95% CI 1.47–2.58, I^2^ = 92%, P < 0.001; Fig. 4a). These findings were consistent with the meta-analysis results with the TyG index analyzed as a continuous variable (three studies [18, 22, 25], HR per 1-unit increment of the TyG index: 1.39, 95% CI 1.18–1.64, I^2^ = 89%, P < 0.001; Fig. 4b). The sensitivity analyses by excluding one study at a time showed similar results (HRs for the TyG index analyzed as a categorical variable: 1.84–2.08, all P < 0.05; HRs for the TyG index analyzed as a continuous variable: 1.31–1.50, all P < 0.05). Moreover, the pooled results of three studies [18, 19, 23] showed that the participants with the highest TyG index category had a significantly increased risk of stroke during follow-up compared to those with the lowest TyG category (HR: 1.26, 95% CI 1.23–1.29, I^2^ = 0%, P < 0.001; Fig. 5).

### Publication bias

**Fig. 6:**
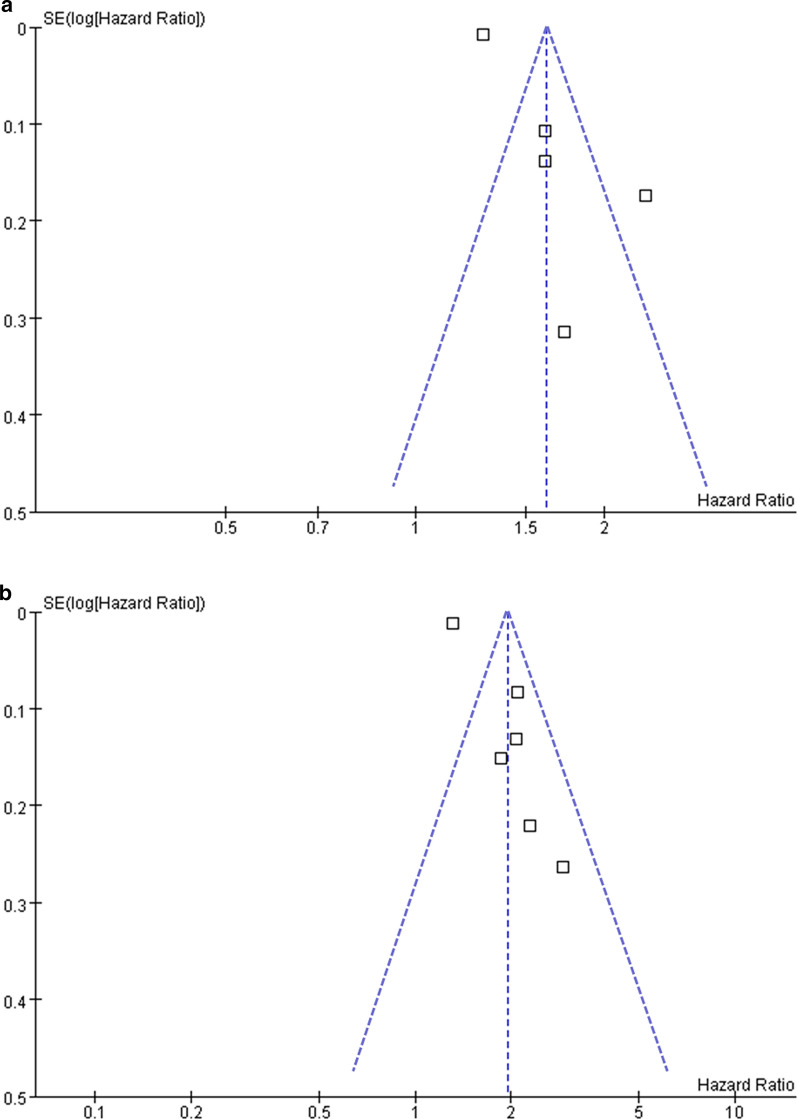
Funnel plots for the publication bias underlying the meta-analysis of the association between the TyG index analyzed as a categorical variable and ASCVD and CAD. **a** Funnel plots for the meta-analysis of the TyG index and ASCVD risk. **b** Funnel plots for the meta-analysis of the TyG index and CAD risk

The funnel plots depicting the association between the serum TyG index analyzed as a categorical variable and ASCVD and CAD are shown in Fig. 6. The funnel plots were symmetric on visual inspection, suggesting a low risk of publication bias. Publication bias for the meta-analysis of the other outcomes was difficult to estimate because a limited number of datasets was included. In addition, Egger’s regression tests were unable to perform, since fewer than ten datasets were available for each outcome.

## Discussion

In this meta-analysis of cohort studies, compared to patients with the lowest TyG index category, those with the highest category were independently associated with an increased incidence of ASCVDs, CAD, and stroke. For ASCVDs, subgroup analyses showed that the association between the TyG index and the subsequent incidence of an ASCVD was not significantly affected by the age, sex, or diabetic status of the participants. Moreover, meta-analysis with the TyG index analyzed as a continuous variable also showed that a higher TyG index at baseline was independently associated with an increased risk of the subsequent incidence of ASCVD or CAD. Taken together, these results suggested that a higher TyG index may be an independent predictor of an increased risk of ASCVD incidence in a population without ASCVDs at baseline.

To the best of our knowledge, this study is the first meta-analysis to summarize the association between the TyG index and the ASCVD incidence in a general population without ASCVDs at baseline. Only cohort studies were included; therefore, the potential recall bias associated with studies having a cross-sectional design was avoided. In addition, only studies with multivariate analyses were included. The results indicated a potential independent association between a higher TyG index and an increased risk of ASCVDs. Moreover, meta-analyses were performed separately with the TyG index analyzed as a categorical variable as well as a continuous variable, and similar results were obtained, thus confirming the robustness of the findings. Furthermore, multiple sensitivity and subgroup analyses were performed to confirm the stability of the findings, which were not driven by a single study or affected by participant characteristics such as age, sex, or diabetic status. Pathophysiologically, the current results reflect the previously suggested role of insulin resistance in the pathogenesis of atherosclerosis [8, 10]. It has been hypothesized that insulin resistance is associated with persistent, low-degree inflammation [33, 34], which is considered to play a key role in the pathogenesis of ASCVDs [9]. Besides, insulin resistance may directly lead to endothelial dysfunction [35], a key pathophysiological process in the initiation and progression of atherosclerosis. Additionally, insulin resistance has been associated with increased activity of the sympathetic nervous system [36] and impaired cardiac autonomic function [37], which have been implicated in the pathogenesis of ASCVDs.

The current study supports the potential of the TyG index to be used as an indicator of ASCVD risk in a general population without ASCVDs. Methodologically, the TyG index can be easily calculated in real-world clinical practice based on routine blood biochemical tests in a cost-effective manner. A previous study has shown that the TyG index is highly sensitive (96.5%) and specific (85.0%) for the detection of insulin resistance, compared to the hyperinsulinemic-euglycemic clamp test [38]. Furthermore, the TyG index has been demonstrated to confer a better performance than homeostatic model assessment for measuring insulin resistance [39]. However, further studies are needed to determine whether the addition of the TyG index to conventional ASCVD risk prediction tools, such as the Framingham risk score, can improve the predictive efficacy in the general population.

Despite the above strengths and potential clinical implications, this meta-analysis has some limitations that should be considered when interpreting the results. First, a limited number of studies was available for this meta-analysis, and significant heterogeneity was detected among them. Additional studies are needed to determine whether other study characteristics can affect the results, such as ethnicity and comorbidities of the participants, follow-up duration, and concurrent medications. In addition, the sample sizes of the included studies varied significantly. For example, for the outcome of stroke, the weight of the study by Hong 2000 is significantly larger than the other two, and the result of the meta-analysis was mainly driven by the result of this study. Second, it remains unknown whether the association between the TyG index and an increased risk of ASCVDs is linear and what the optimal cut-off value of the TyG index is for the prediction of future risk of ASCVDs. Third, residual confounding factors possibly affecting the association between the TyG index and the ASCVD risk, such as the dietary and nutritional factors that may affect the TyG index, could not be excluded [40]. Finally, this meta-analysis was based on cohort studies; thus, a causative association between the TyG index and the incidence of ASCVDs cannot be implied.

## Conclusions

In conclusion, the existing evidence from cohort studies suggests that a higher TyG index may be an independent predictor of ASCVD risk in people without ASCVDs at baseline. Due to the convenience of measuring the TyG index in clinical settings, future studies are needed to determine whether incorporation of the TyG index on top of the ASCVD risk prediction tools currently used can improve their predictive efficacies.

## Data Availability

All data generated or analysed during this study are included in this published article.

## Abbreviations

ASCVDs: Atherosclerotic cardiovascular diseases
CAD: Coronary artery disease
TyG: Triglyceride–glucose
PAD: Peripheral artery disease
HRs: Hazard ratios
Cis: Confidence intervals
